# Gene Expression Analysis of Aggressive Adult Xp11.2 Translocation Renal Cell Carcinoma at Clinical Stage T1N0M0 to Identify Potential Prognostic and Therapeutic Biomarkers

**DOI:** 10.3390/biomedicines10020321

**Published:** 2022-01-29

**Authors:** Jee Soo Park, Myung Eun Lee, Won Sik Jang, Jongchan Kim, Se Mi Park, Won Sik Ham

**Affiliations:** 1 Department of Urology and Urological Science Institute, Yonsei University College of Medicine, Seoul 03722, Korea; jsparkysmed@gmail.com (J.S.P.); lme0228@yuhs.ac (M.E.L.); sindakjang@yuhs.ac (W.S.J.); lumpakcef@yuhs.ac (J.K.); mistrie694@yuhs.ac (S.M.P.); 2 Department of Urology, Sorokdo National Hospital, Goheung 59562, Korea; 3 Department of Urology, Yongin Severance Hospital, Yonsei University Health System, Seoul 03722, Korea

**Keywords:** renal cell carcinoma, Xp11.2, translocation, *TFE3*, biomarker, *PIK3R2*

## Abstract

Xp11.2 translocation renal cell carcinoma (tRCC), involving transcription factor E3 (*TFE3*) gene fusions, is a rare and aggressive RCC variant when present in adults and has been recently recognized as a unique entity in RCC. Biomarkers and treatment guidelines do not exist for patients with aggressive Xp11.2 tRCC. The aim was to identify and evaluate therapeutic biomarkers for aggressive Xp11.2 tRCC. RNA sequencing was performed using formalin-fixed, paraffin-embedded tissues from 11 adult patients with clinical T1N0M0 Xp11.2 tRCC, including three patients with aggressive characteristics (recurrence or cancer-specific death after nephrectomy). Thirty genes were differentially expressed between the aggressive and non-aggressive groups, even after adjustment, and were associated with KEGG pathways related to the aggressiveness of Xp11.2 tRCC. *PIK3R2*, involved in various KEGG pathways, including the PI3K/AKT/mTOR pathway, was overexpressed in the Xp11.2 tRCC cell lines UOK120 and UOK146. The PI3K pathway inhibitor LY294002 showed a significant therapeutic benefit. This study provides the first candidate biomarker, *PIK3R2*, for aggressive clinical T1N0M0 Xp11.2 tRCC. Furthermore, this study is the first to recommend a targeted drug, LY294002, for aggressive Xp11.2 tRCC based on the molecular pathophysiology.

## 1. Introduction

Renal cell carcinoma (RCC) involving translocations at Xp11.2, referred to as Xp11.2 translocation RCC (tRCC), is a rare subtype and has gained significant clinical interest in the last two decades [1,2], after its inclusion as a new distinct entity in the 2004 World Health Organization (WHO) classification of renal tumors [3]. tRCC has been subclassified as belonging to the microphthalmia transcription factor-associated family tRCC in the updated 2016 WHO classification based on improvements in genetic profiling technology [4]. Xp11.2 tRCC is predominantly reported in children, accounting for about one-third of pediatric RCC cases [5,6]. However, tRCC is rare in adults, accounting for 1.6–5.0% for adult RCC cases [7,8]. This low incidence might be due to morphological overlap with clear cell RCC (ccRCC) and papillary RCC, resulting in its misdiagnosis [8,9].

Numerous fusion partners in Xp11.2 tRCC have been identified. *PRCC*, *ASPL*/*ASPSCR1*, and *SFPQ* are the most common partners, and many novel genes are still being discovered [9,10]. The *ASPL*/*ASPSCR1-TFE3* fusion type is associated with unfavorable outcomes [7] and shares the same chromosomal breakpoint as alveolar soft part sarcoma, which is refractory to chemotherapy [11]. The *PRCC-TFE3* fusion type is associated with nodal involvement; however, patients can be disease-free for at least 3 years after lymph node dissection [7].

There are only a few case reports and observational studies of systemic therapies for advanced Xp11.2 tRCC, most of which focused on sunitinib [1]. Systemic treatment guidelines for advanced Xp11.2 tRCC are currently unavailable owing to the rarity of the disease as well as the poor understanding of its molecular pathophysiology [1]. In this study, tissue samples were analyzed to develop molecular biomarkers for aggressive Xp11.2 tRCC and evaluated the value of these biomarkers for determining the appropriate systemic therapy.

## 2. Materials and Methods

### 2.1. Patients and Tissues

For this retrospective study, data were gathered from patients with Xp11.2 tRCC who underwent radical nephrectomy between January 2008 and December 2018 and had available formalin-fixed, paraffin-embedded (FFPE) tissues. Tumors sized ≤7 cm (clinical T1 stage) without synchronous metastasis on images (N0M0 stage) were included. Patients with Xp11.2 tRCC pathologically confirmed by TFE3 immunohistochemistry staining and fluorescence in situ hybridization (FISH) assay were included. Immunohistochemistry (IHC) was performed on FFPE sections with a TFE3 antibody; tumors were considered TFE3-positive when labeling was moderate to strong (2+ to 3+) in more than 10% of cells. Tumors with non-diffuse (<10% of cells) and weak labeling (1+) were excluded. A dual-color, break-apart FISH assay was performed with samples from patients who had positive TFE3-IHC results on FFPE tissue microarray slides. Patients were divided into aggressive and non-aggressive Xp11.2 tRCC groups. An aggressive tumor was defined as a tumor exhibiting recurrence or cancer-specific death.

### 2.2. Tissue Preparation

A urologic pathologist reviewed all FFPE sections of Xp11.2 tRCC patients, which were obtained from the archives of the Department of Pathology at Yonsei University College of Medicine (Seoul, Korea). Identification of non-tumor elements was based on hematoxylin and eosin-stained slides. The samples were cut into 20 μm sections and transferred to an extraction tube.

### 2.3. RNA Extraction and Sequencing

Briefly, RNA was extracted from 20 μm sections of FFPE tissues using an FFPE RNeasy Kit (Qiagen, Gaithersburg, MD, USA), and 100 ng of RNA was used to construct a sequencing library using a SureSelectXT RNA Direct Library Preparation Kit (Agilent Technologies, Santa Clara, CA, USA) according to the manufacturer’s protocols.

The sequencing libraries were quantified using a Kapa Biosystems Library Quantification Kit for Illumina Sequencing platforms according to the quantitative polymerase chain reaction (qPCR) Quantification Protocol Guide (Kapa Biosystems, Wilmington, MA, USA; Cat# KK4854) and were qualified using TapeStation D1000 ScreenTape (Agilent Technologies, Santa Clara, CA, USA; Cat# 5067-5582). Indexed libraries were then subjected to paired-end sequencing (2 × 101 bp) using the NovaSeq 6000 system (Illumina, San Diego, CA, USA) by Macrogen (Seoul, Korea).

### 2.4. RNA Sequencing (RNA-Seq) Analysis and Identification of Differentially Expressed Genes

Paired-end sequencing reads from cDNA libraries (101 bp) were generated, and the trimmed reads were aligned to the human reference genome, hg19 (GRCh37), using HISAT (v2.1.0). The transcriptome assembly of known transcripts, novel transcripts, and alternative splicing transcripts was processed.

Transcript abundance and gene expression levels were calculated as read counts (i.e., number of reads mapped to a gene) or fragments per kilobase of exon per million fragments mapped (FPKM) per sample. To identify differentially expressed genes (DEGs) between aggressive and non-aggressive Xp11.2 tRCC groups, genes with more than one sample with a zero read count were pre-filtered. Expression levels of 35,993 RefSeq genes and 19,594 genes were retained.

For DEGs, an unsupervised hierarchical clustering analysis was performed, with the complete linkage method and Euclidean distances as a measure of similarity. A principal component analysis (PCA) was performed to reduce the dimensionality of the dataset by transforming it into a new set of variables summarizing the data features. All data analyses and the visualization of DEGs were conducted using R.3.5.1 (available online: www.r-project.org (accessed on 23 December 2021)).

### 2.5. Fusion Type Analysis

Fusion genes from RNA-Seq data were identified using FusionCatcher and Arriba. FusionCatcher searches for novel/known somatic fusion genes, translocations, and chimeras from RNA-Seq data. In brief, FusionCatcher filters and trims low-quality reads and then finds fusion junctions with at least two genes (both in the ENSEMBL database) using known exon and intron information. Arriba is a command-line tool for the detection of gene fusions from RNA-Seq data. It is based on the ultrafast STAR aligner.

### 2.6. KEGG Analysis

A KEGG (Kyoto Encyclopedia of Genes and Genomes) pathway analysis of DEGs was performed based on the KEGG pathway (available online: https://www.genome.jp/kegg/ (accessed on 26 December 2021)) database. A sorted heatmap of KEGG enrichment results was generated to visualize significant pathways filtered by a *p*-value of <0.05, as determined by the Fisher’s exact test.

### 2.7. Xp11.2 tRCC Cell Lines

tRCC cell lines with the two most common gene fusions, *ASPL-* and *PRCC-TFE3*, were obtained. UOK120 and UOK146 were previously derived from tumors excised from patients with Xp11.2 tRCC who were treated at the National Cancer Institute (NCI, Bethesda, MD, USA) and harbored *PRCC-TFE3* gene fusions [12]. The UOK120 and UOK146 lines were obtained from and authenticated by the NCI (Bethesda, MD, USA). S-TFE, which was established from patients with tRCC with *ASPL-TFE3* gene fusions [13], was obtained from the Riken Cell Bank (Tsukuba, Japan). These cells were cultured in Dulbecco’s modified Eagle’s medium or Roswell Park Memorial Institute 1640 medium, supplemented with 10% fetal bovine serum (FBS; Gibco; Thermo Fisher Scientific, Waltham, MA, USA) and 1% penicillin–streptomycin (Sigma-Aldrich, St. Louis, MO, USA), at 37 °C in a humidified atmosphere containing 5% CO_2_.

### 2.8. Treatment Regimens

The anticancer drugs sunitinib, sorafenib, everolimus, and LY294002 (a PI3K pathway inhibitor) were purchased from Selleckchem (Houston, TX, USA).

### 2.9. Cell Proliferation Assay

Cells were seeded at 3000–5000 cells per well in 96-well culture plates. The next day, the cells were treated with anticancer drugs at the desired concentration in culture media. At 24–72 h after treatment, cell viability was assessed by a CCK-8 assay (Dojindo Laboratories, Kumamoto, Japan), in accordance with the manufacturer’s instructions. Absorbance was detected at 450 nm using a VersaMax Microplate Reader (Molecular Devices, Sunnyvale, CA, USA).

### 2.10. Reverse Transcription-qPCR Analysis

To detect *PIK3R2* expression, total RNA was extracted using the RNeasy Mini Kit (Qiagen Inc., Valencia, CA, USA). A complementary DNA template was synthesized from 1 μg of RNA using iNtRon Maxime RT PreMix (iNtRON Biotechnology, Seongnam, Korea), and qPCR was performed using the StepOnePlus Real-Time PCR system (Applied Biosystems, Foster City, CA, USA). The reaction conditions were as follows: predenaturation at 95 °C for 10 min, denaturation at 95 °C for 10 s, annealing at 60 °C for 20 s, and extension at 72 °C for 34 s, with a total of 40 cycles. U6 small nuclear RNA was used as the internal reference. The qPCR data were analyzed using the 2^−ΔΔCT^ method. These procedures were repeated three times.

### 2.11. Wound-Healing Assay

Cells were cultured to confluence in 6-well plates. When the cells reached 100% confluence, a scratch was produced by a sterile pipette tip. The cells were rinsed two times in phosphate-buffered saline and treated with 10 µM LY294002 in culture media. Images of cells were obtained under an inverted microscope at 0 and 24 h. ImageJ was used to calculate the healing area, and the healing rate (expressed as a percentage) was calculated as follows: (initial scratch width—existing scratch width)/initial scratch width × 100.

### 2.12. Invasion Assay

In vitro Matrigel invasion assays were performed using the CytoSelect ™ 24-Well Cell Invasion Assay Kit (Cell Biolabs, San Diego, CA, USA) following the manufacturer’s instructions. Cells were treated with phosphate-buffered saline or LY294002 (10 µM) and plated on the upper chamber (2–5 × 10^4^ cells/well) using serum-free medium. For the assays, 10% FBS-containing medium was placed in the lower chambers as a chemoattractant. After incubation at 37 °C for 24 h, invading cells on the bottom surface of the membrane were stained with crystal violet, and cells passing through the Matrigel in each group were counted as an indicator of invasion ability.

### 2.13. Western Blot Analysis

To detect PIK3R2 expression, proteins were extracted with radioimmunoprecipitation assay buffer (Cell Signaling Technology, Danvers, MA, USA), and the amount of protein was determined using a bicinchoninic acid protein assay kit (Pierce, Rockford, IL, USA). Equal amounts of denatured proteins were separated by sodium dodecyl sulfate–polyacrylamide gel electrophoresis, transferred to a polyvinylidene fluoride membrane, and blocked with 5% bovine serum albumin in tris-buffered saline with Tween 20. Samples were incubated with antibodies to PIK3R2 (1:2000; Santa Cruz Biotechnology, Dallas, TX, USA) and β-actin (1:5000, Cell Signaling Technology) at 4 °C overnight and subsequently with horseradish peroxidase-conjugated secondary antibody (Santa Cruz Biotechnology). To detect reactive bands, the membranes were examined using the ECL Prime Western Blotting Detection System (GE Healthcare, Amersham, UK) and LAS-3000 (Fujifilm, Tokyo, Japan).

### 2.14. Statistical Analysis

Statistical analyses were performed using GraphPad Prism version 8.0 (GraphPad Software, Inc., La Jolla, CA, USA) and SPSS version 23.0 (IBM Corp., Armonk, NY, USA). All results are expressed as means ± standard deviation (s.d.) unless otherwise indicated. The Student’s *t*-test was used for data that followed a normal distribution, as determined by the Shapiro–Wilk normality test. If the dataset did not follow a normal distribution, the Mann–Whitney U test was used. All statistical tests were two-tailed, and *p* < 0.05 was considered significant.

## 3. Results

### 3.1. Baseline Characteristics

Clinicopathologic characteristics of patients with clinical T1N0M0 Xp11.2 tRCC at presentation (*n* = 11) are summarized in Table 1. The mean age and tumor size were 45.1 ± 18.2 years and 4.9 ± 1.7 cm, respectively. Among 11 patients, three patients had recurrence or cancer-specific death and were assigned to the aggressive Xp11.2 tRCC group. Age, tumor size, or World Health Organization/International Society of Urologic Pathologists grade did not differ significantly between patients with aggressive Xp11.2 tRCC and those with non-aggressive Xp11.2 tRCC. All three patients in the aggressive group exhibited tumor recurrence after nephrectomy within a mean follow-up period of 20.7 months. One patient exhibited cancer-specific death after 11.0 months of follow-up. Survival time did not differ significantly between the aggressive and non-aggressive Xp11.2 tRCC groups (*p* = 0.290).

### 3.2. RNA-Seq Analysis

The RNA-seq analysis generated 10,256 × 10^6^ base pairs (bp) from Xp11.2 tRCC cases with aggressive characteristics and 10,521 × 10^6^ bp from Xp11.2 tRCC cases without aggressive characteristics. In total, 101 × 10^6^ reads from Xp11.2 tRCC cases with aggressive characteristics and 104 × 10^6^ reads in Xp11.2 tRCC cases without aggressive characteristics were mapped. There were no differences in the number of reads between the two groups, and 19,594 genes were identified.

### 3.3. DEGs, Fusion Types, and KEGG Pathway Enrichment

**Figure 1:**
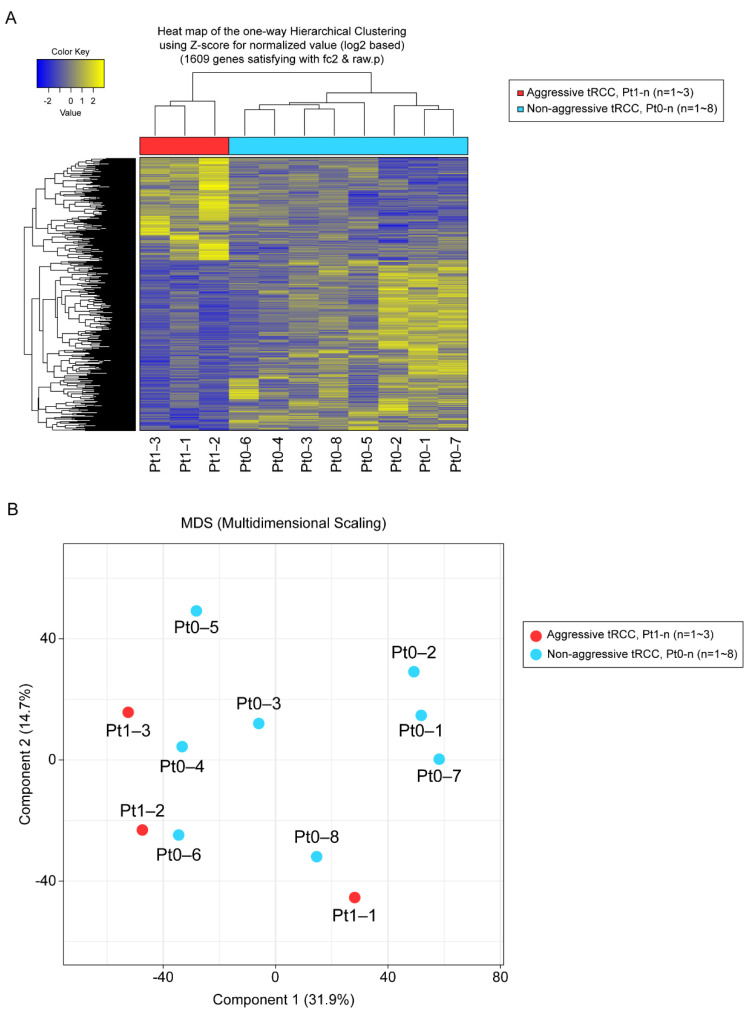
(**A**) Unsupervised hierarchical clustering analysis (yellow, high relative expression; blue, low relative expression) of patients with aggressive Xp11.2 translocation renal cell carcinoma (tRCC). (**B**) Principal component analysis. The first and second principal component scores in patients with aggressive Xp11.2 tRCC (*n* = 3, red) versus matched patients with non-aggressive Xp11.2 tRCC (*n* = 8, blue) are plotted.

In total, 1609 genes were differentially expressed with fold changes ≥2 and *p* < 0.05, including 603 upregulated genes and 1006 downregulated genes in Xp11.2 tRCCs with aggressive characteristics compared to matched Xp11.2 tRCCs without aggressive characteristics (Figure 1). In total, 91 genes were strongly upregulated or downregulated in patients with aggressive tRCCs and retained significance even after adjustment. Among these, 77 genes were protein-coding genes, and four genes were long non-coding RNAs; the others were pseudogenes. Clear clustering was observed, distinguishing between the aggressive and non-aggressive Xp11.2 tRCC groups, as determined by PCA (Figure 1).

Among 11 patients, fusion types were identified for five patients. Among patients with aggressive tRCC, Pt1-2 and Pt1-3 harbored the *ASPL-TFE3* gene fusion and *RBM10-TFE3* gene fusion, respectively. For patients with non-aggressive tRCC, Pt0-4, Pt0-6, and Pt0-8 harbored the *SFPQ-TFE3* gene fusion.

**Figure 2:**
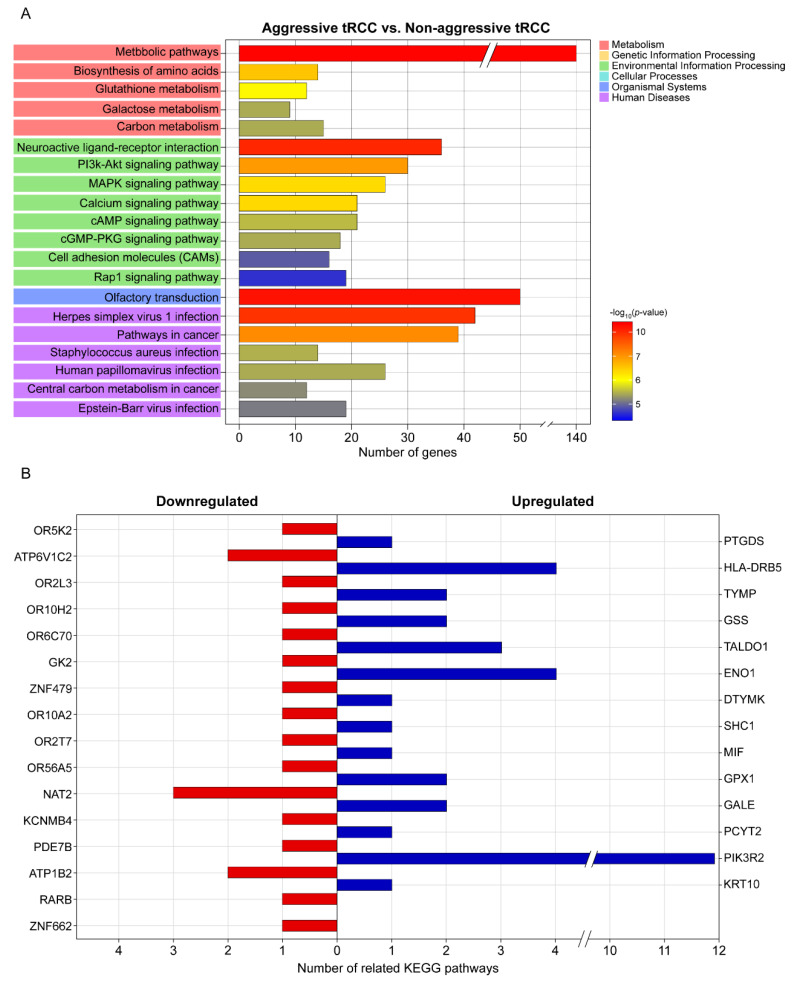
KEGG pathway analysis. (**A**) Top 20 significant KEGG pathways and their functional classification by five main categories (A, cellular processes; B, environmental information processing; C, genetic information processing; D, metabolism; and E, organismal systems). The *x*-axis indicates the number of genes annotated under the indicated pathway. The *y*-axis indicates the names of KEGG pathways. The bar indicates the −log10 (*p*-value). (**B**) Genes significantly associated with the aggressiveness of Xp11.2 translocation renal cell carcinoma and the number of related KEGG pathways for each gene. KEGG, Kyoto Encyclopedia of Genes and Genomes.

A KEGG pathway enrichment analysis revealed that the DEGs were mainly enriched in metabolic pathways, olfactory transduction, neuroactive ligand–receptor interaction, herpes virus 1 infection, pathways in cancer, and the PI3K-Akt signaling pathway (Figure 2A). The top 20 significant pathways associated with the aggressiveness of Xp11.2 tRCC (Figure 2A) involved genes that were significantly associated with the aggressiveness of Xp11.2 tRCC, including 14 upregulated and 16 downregulated genes (Table 2 and Figure 2B). Among the 30 genes that were significantly associated with the aggressiveness of Xp11.2 tRCC, *PIK3R2* was involved in the most known pathways (i.e., 11 pathways). PIK3R2 expression levels in aggressive Xp11.2 tRCC (27.44 ± 4.87 FPKM) were significantly higher than those in non-aggressive Xp11.2 tRCC (14.15 ± 2.45 FPKM) (adjusted-*p* > 0.04 by the Benjamini–Hochberg algorithm).

### 3.4. PIK3R2 Expression in tRCC Cell Lines

**Figure 3:**
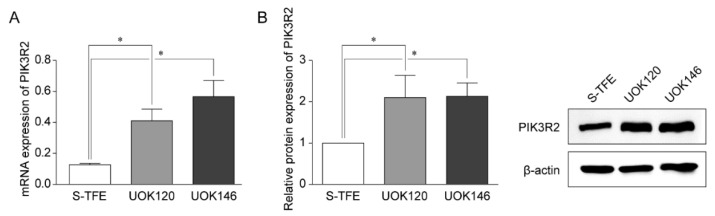
Expression of PIK3R2 in Xp11.2 translocation renal cell carcinoma (tRCC) cells. (**A**) Expression of *PIK3R2* mRNA in tRCC cells detected by quantitative real-time polymerase chain reaction. (**B**) Relative PIK3R2 protein expression. Western blotting was used to detect the expression of PIK3R2 protein in tRCC cells. * *p* < 0.01.

Reverse transcription-qPCR and Western blotting were performed to detect the expression of PIK3R2 in tRCC cell lines (S-TFE, UOK120, and UOK146) (Figure 3). PIK3R2 expression levels were significantly higher in UOK120 and UOK146 than in S-TFE (*p* < 0.05, Figure 3).

### 3.5. Effects of Various Treatments on tRCC Cell Proliferation

After treatment with LY294002, sunitinib, sorafenib, and everolimus in Xp11.2 tRCC cell lines (S-TFE, UOK120, and UOK146), the viability of each cell line was measured every 24 h until 72 h post-treatment.

**Figure 4:**
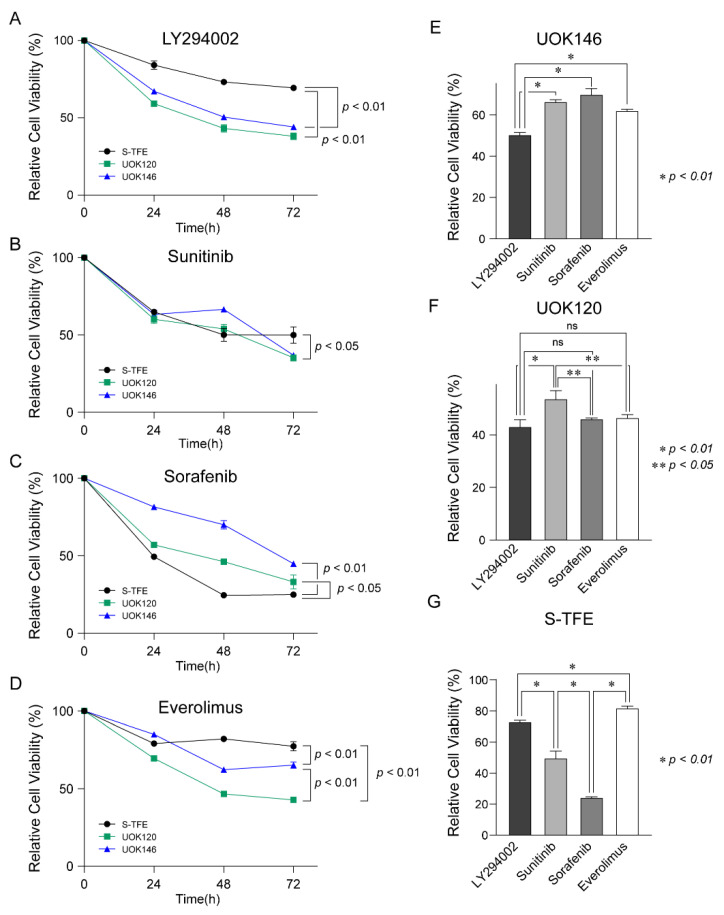
Therapeutic efficacy of LY294002, sunitinib, sorafenib, and everolimus in Xp11.2 translocation renal cell carcinoma (tRCC) cells. Proliferation curves for Xp11.2 tRCC cells treated with (**A**) LY294002, (**B**) sunitinib, (**C**) sorafenib, or (**D**) everolimus for 24, 48, and 72 h. (**E**) UOK146, (**F**) UOK120, and (**G**) S-TFE cell viability after LY294002, sunitinib, sorafenib, and everolimus treatment for 48 h. * *p* < 0.01, ** *p* < 0.05.

After treatment with LY294002, cell viability was lower for UOK120 and UOK146 than S-TFE (*p* < 0.01) (Figure 4A). Sunitinib also decreased the proliferation rates more substantially for UOK120 and UOK146 than for S-TFE (*p* < 0.05) (Figure 4B); however, the effect was not as highly significant as that of LY294002. In contrast, sorafenib resulted in a greater reduction in the rate of proliferation of S-TFE than UOK120 (*p* < 0.05) or UOK146 (*p* < 0.01) (Figure 4C). After treatment with everolimus, viability was significantly lower for UOK120 and UOK146 than for S-TFE (*p* < 0.05) (Figure 4D).

As shown in Figure 4E, UOK146 cell viability was significantly lower following LY294002 treatment than following sunitinib, sorafenib, or everolimus treatment (*p* < 0.01). In terms of UOK120 cell viability, LY294002 treatment was more effective than sunitinib treatment (*p* < 0.01); sorafenib and everolimus also resulted in significantly lower cell proliferation rates than those observed after sunitinib treatment (*p* < 0.05) (Figure 4F). The proliferation of S-TFE was more effectively reduced by sorafenib than by other treatments (*p* < 0.01), and sunitinib was the second most effective treatment (Figure 4G).

### 3.6. Effects of LY294002 on the Migration and Invasion Ability of tRCC Cell Lines

**Figure 5:**
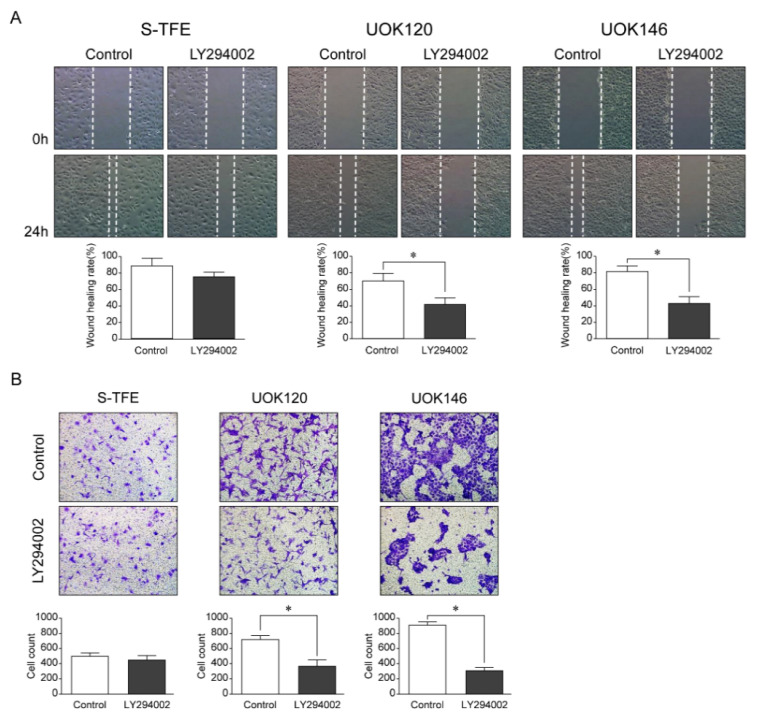
Effects of LY294002 (a PI3K pathway inhibitor) on aggressive Xp11.2 translocation renal cell carcinoma (tRCC) cells. (**A**) The cell scratch test was used to detect the effect of PI3K inhibitor treatment on the migration of Xp11.2 tRCC cells. The migration ability of UOK120 and UOK146 was significantly inhibited by treatment with 10 µM LY294002 (×10). (**B**) An invasion assay was used to detect the effect of PI3K inhibitor treatment on the invasion of Xp11.2 tRCC cells. The invasive ability of UOK120 and UOK146 was significantly inhibited by treatment with 10 µM LY294002 (×10). * *p* < 0.01.

After LY294002 treatment, the migration ability and number of invasive cells of UOK120 and UOK146 were significantly lower than those of S-TFE (*p* < 0.01) (Figure 5).

## 4. Discussion

The therapeutic biomarker *PIK3R2* was identified for aggressive Xp11.2 tRCC, and the efficacy of a novel therapy (i.e., the PI3K pathway inhibitor LY294002) was evaluated according to this biomarker. This study is the first to suggest a targeted agent for Xp11.2 tRCC treatment based on a therapeutic biomarker determined by scientific rationale, with an understanding of the molecular pathophysiology and verification in several tRCC cell lines.

Owing to the low prevalence of Xp11.2 tRCC, treatment guidelines are not available [14]. Xp11.2 tRCC is mainly diagnosed in children, and its diagnosis is rarely reported in the adult population [7,9,15,16]. Although its prevalence in adults is low, the presentation is more advanced and aggressive [17,18,19]. The optimal treatment option for aggressive Xp11.2 tRCC remains undetermined [7]. Accordingly, guidelines for systemic therapy in Xp11.2 tRCC are urgently needed. Although several targeted agents have been approved for the treatment of advanced RCC and are used in all RCC subtypes, clinical trials, for the most part, have focused on the most common subtype, ccRCC [20]. Furthermore, no studies have evaluated the therapeutic efficacies of current systemic therapies in Xp11.2 tRCC cell lines. This is the first study to validate the therapeutic efficacy of currently available targeted agents in Xp11.2 tRCC cell lines.

No clinical trials have suggested the use of targeted agents in Xp11.2 tRCC, and most are based on observational studies, mainly using sunitinib [1]. Choueiri et al. have reported the effect of anti-vascular endothelial growth factor (VEGF) therapies (sunitinib, sorafenib, and monoclonal anti-VEGF antibodies) in 15 patients with metastatic Xp11.2 tRCC, with objective response rates of 66.7% [20]. Additionally, Solano et al. reported a case demonstrating the therapeutic efficacy of pazopanib, a similar anti-VEGF agent [1]. Alternatively, some studies have suggested that the mammalian target of rapamycin (mTOR) inhibitors is a treatment option for Xp11.2 tRCC [21]. Malouf et al. reported an objective response rate of 33.0% for an mTOR inhibitor in patients with metastatic Xp11.2 tRCC [22]. Additionally, the therapeutic benefits of mTOR inhibitors have been demonstrated in a patient with Xp11.2 tRCC who did not show a favorable response to first-line VEGF inhibitor treatment [21]. Based on the current literature, targeted molecular therapies, including VEGF receptor inhibitors and mTOR inhibitors, have therapeutic advantages over immunotherapy in Xp11.2 tRCC [23]. We evaluated and compared the therapeutic efficacy of sunitinib, sorafenib, and everolimus, which are known to be effective in treatments of Xp11.2 tRCC in addition to LY294002, the PI3K pathway inhibitor, which targets the overexpression of PIK3R2 in aggressive Xp11.2 tRCC patients.

In the present study, sunitinib and sorafenib, which are VEGFR inhibitors, demonstrated a significantly better efficacy in the S-TFE cell line, which has the *ASPL-TFE3* gene fusion, than in the UOK120 and UOK146 cell lines, which have the *PRCC-TFE3* gene fusion. In contrast, LY294002 and mTOR inhibitors demonstrated better therapeutic efficacy in the UOK120 and UOK146 cell lines than in the S-TFE cell line. In addition to the difference in fusion genes, UOK120 and UOK146 cell lines have elevated levels of PIK3R2, which is the potential target of LY294002, unlike the S-TFE cell line. Therefore, LY294002 has the greatest therapeutic benefit in UOK120 and UOK146 cell lines. Additionally, PI3K/AKT/mTOR signaling pathway dysregulation is frequently identified in patients with RCC, and mTOR inhibitors, including everolimus and temsirolimus, are effective in metastatic ccRCC [24]. mTOR inhibitors also have therapeutic effects in adult patients with Xp11.2 tRCC with aggressive courses [21].

Although the mechanism underlying the effects of VEGF-targeted therapies in Xp11.2 tRCC is largely unknown, a few studies have provided insight into the mechanism underlying the effects of mTOR inhibitors [25]. In particular, phosphorylated 4EBP1 acts as a critical factor for integrating upstream oncogenic signals in the PI3K/AKT/mTOR signaling pathway and drives proliferative downstream signaling [25]. Our results also revealed a significant association of the PI3K/AKT/mTOR signaling pathway with aggressive Xp11.2 tRCC. Dysregulation of the PI3K/AKT/mTOR signaling pathway, which contributes to various biological processes, such as apoptosis, metabolism, cell proliferation, and cell growth, frequently occurs in cancer [26]. In RCC, the PI3K/AKT/mTOR signaling pathway serves an important role in tumorigenesis [24]. Song et al. reported that a decrease in the expression of the target gene *PIK3R2* influences the PI3K/AKT/mTOR signaling pathway, thereby suppressing the proliferation, migration, and invasive abilities of a human lung cancer cell line [26]. *PIK3R2*, which encodes the p85ß regulatory subunit of PI3K (phosphatidylinositol-3-kinase), is frequently overexpressed in melanoma, breast, and squamous cell lung carcinoma, and its expression level increases during tumor progression [27]. The role of *PIK3R2* as an oncogene has not been investigated in RCC, especially in tRCC. Our results provide the first evidence for the overexpression of PIK3R2 in aggressive Xp11.2 tRCC and show that suppressing PIK3R2 by LY294002 in Xp11.2 tRCC cell lines eventually leads to a decrease in proliferation, migration, and invasion abilities, similar to observations in lung cancer cell lines. Additionally, everolimus demonstrated a better therapeutic efficacy than sunitinib in the UOK120 and UOK146 cell lines in the present study. This can be explained by the overexpression of PIK3R2 in these cell lines, since the activation of PIK3R2 increases PI3K/AKT/mTOR signaling pathway activity, leading to the overexpression of mTOR.

In exploring the association between the fusion type and oncological outcomes, Komai et al. demonstrated different biological behaviors among different fusion types of Xp11.2 tRCC [7]. In this previous study, four patients, including two with the *ASPL-TFE3* gene fusion, had an unfavorable prognosis, and one patient with the *PRCC-TFE3* gene fusion with nodal involvement remained disease free for 3 years after lymph node dissection [7]. The two patients with the *ASPL-TFE3* gene fusion both developed visceral metastases, and one patient died of the disease [7]. The *ASPL-TFE3* fusion seems to have a worse prognosis, with frequent lymph node metastasis; however, it is not clear whether the fusion partner has prognostic value. In the present study, although not all fusion types were identified, associations between those that were identified and the prognosis were consistent with previous results. For patients with aggressive tRCC, the *ASPL-TFE3* gene fusion and *RBM10-TFE3* gene fusion were found. Along with *ASPL-TFE3*, the *RBM10-TFE3* gene fusion is also associated with an aggressive course. Kato et al. reported that among 10 patients, two patients died, and three patients developed recurrence [28]. We detected *SFPQ-TFE3* gene fusion in three patients with non-aggressive tRCC. Argani et al. reported seven cases of *SFPQ-TFE3* gene fusion Xp11.2 tRCC, where all cases were confined to the kidney, and only one case showed recurrence [29]. The *SFPQ-TFE3* gene fusion seems to have a more favorable prognosis than the *ASPL-TFE3* gene fusion [29], which is consistent with our findings.

In terms of the *PRCC-TFE3* gene fusion, oncological outcomes are controversial. Although Komai et al. reported a favorable prognosis for the *PRCC-TFE3* gene fusion and poor oncological outcomes for the *ASPL-TFE3* gene fusion [7], Argani et al. reported two cases of the *PRCC-TFE3* gene fusion with metastatic disease [29]. In our study, although the *PRCC-TFE3* gene fusion was not identified in patient samples, aggressiveness was associated with the *PRCC-TFE3* gene fusion. The S-TFE cell line had the *ASPL-TFE3* gene fusion, while the UOK120 and UOK146 cell lines had the *PRCC-TFE3* gene fusion and showed *PIK3R2* overexpression, which was associated with the aggressiveness of Xp11.2 tRCC. Therefore, the gene fusion types show a difference in the expression of *PIK3R2.* However, a direct comparison between studies is difficult for a few reasons. First, among four cases in a study by Komai et al., one patient was 15 years old, while other studies excluded patients younger than 18 years [30]. Many studies have reported a difference in oncological outcomes between adults and children [30]; thus, the inclusion of adults and children in one study would cause a selection bias. Second, Komai et al. reported a tumor more than 7 cm in size [7]; in contrast, only cases with tumor sizes <7 cm were included in the present study. Previous studies of the tumor size in Xp11.2 tRCC have revealed that a larger tumor size (*p* < 0.01) is associated with aggressiveness [30]. However, in the present study, the tumor size did not significantly differ between the aggressive and non-aggressive Xp11.2 tRCC groups. This might be because the study population was limited to those with clinical stage T1. Third, previous studies have included cases with nodal metastasis at the time of initial diagnosis, including Komai et al. [7]. Nodal disease without distant metastasis has a favorable short-term prognosis after surgery alone in patients with Xp11.2 tRCC [7,16]. The present study included patients who developed nodal metastasis after surgery, for whom the initial presentation was at the clinical stage T1N0M0. Although it was not within the scope of the present study, future studies are needed to evaluate the correlation between fusion type and biological behavior in patients with Xp11.2 tRCC, including clinical T1N0M0 Xp11.2 tRCC.

The natural history of Xp11.2 tRCC is poorly understood; however, growing evidence indicates that adult Xp11.2 tRCC is clinically aggressive and often presents at an advanced stage, with poor outcomes [31]. Argani et al. reported that 50% of 28 adult Xp11.2 tRCC cases presented with stage 4 disease [9]. Qu et al. reported that 26.7% of patients had recurrence and 30.0% of patients died of cancer in a study of 30 adult patients with Xp11.2 tRCC [31]. Kuthi et al. reported that 33% of patients died from cancer-related causes in a cohort of 28 patients; however, three children were included in this cohort [32]. Similarly, there were three aggressive cases (27.3%; including patients with recurrence or cancer-related death) among the 11 cases in the present study. These data indicate that Xp11.2 tRCC has the same recurrence and mortality rates as ccRCC [33]. Although the clinical behavior of Xp11.2 tRCC has been reported to be more aggressive in adults than in children [31], this might be due to relative indolent course of Xp11.2 tRCC in children.

Xp11.2 tRCCs are typically considered as a malignancy primarily found in pediatric patients, with very low incidence in adult populations [7,34]. However, being recognized as a distinct entity, the prevalence of Xp11.2 tRCC in adult populations is increasing, and its behavior is considered aggressive compared to pediatric onset Xp11.2 tRCC, which has been reported as a benign clinical course [34]. Therefore, a treatment strategy is urgently needed. Based on the molecular understanding of pathophysiology of aggressive Xp11.2 tRCC, we have suggested novel therapy for advanced Xp11.2 tRCC, for which systemic treatment guidelines are currently unavailable. We believe that our study will be the cornerstone of the research on novel therapeutic approaches for Xp11.2 tRCC.

The major strength of the present study was the inclusion of a relatively large sample size, with long-term follow-up periods. However, there were some limitations as well. First, there is the possibility of selection bias. This study only included Korean patients treated at a single institution. However, the results were supported by cancer cell lines developed in the USA and Japan. Second, the RNA-Seq technique has poor sensitivity in detecting fusion genes; thus, the fusion partner genes could not be fully explored [35]. The identification of fusion partner genes associated with PIK3R2 expression should be a focus of future research. Third, international guidelines now suggest the use of immune-based combination treatments as the first-line systemic treatment for metastatic disease, based on the results of a series of randomized phase III trials [36,37]. However, due to the difficulty of developing appropriate humanized mouse models that mimic the human immune system and inferior therapeutic benefit of immunotherapy over VEGF receptor inhibitors and mTOR inhibitors in Xp11.2 tRCC patients, we have not evaluated the therapeutic efficacy of immune-based combination treatments. However, since immunotherapy is emerging as the main treatment for metastatic RCC, future studies will incorporate immunotherapy by using humanized mouse models.

## 5. Conclusions

This study is the first to report a therapeutic biomarker for aggressive Xp11.2 tRCC in clinically localized T1 stage, where the prognosis is generally good. PIK3R2, which is highly expressed in patients with aggressive Xp11.2 tRCC, is a biomarker for the use of a novel therapy, LY294002. More data are needed to determine the best treatment methods and prognostic markers for patients with Xp11.2 tRCC, including international multicenter studies with large sample sizes.

## Figures and Tables

**Table 1:** Clinicopathological characterization of the study population.

Aggressive (Y/N)	Patient ID	Sex	Age (year)	Tumor Size (cm)	WHO/ISUP Grade	Outcome ^a^	Recurrence-Free Time ^b^ (m)	Recurrence Site	Death (Y/N)	Survival Time ^c^ (m)
N	Pt0-1	F	21	2.5	2	ned			N	111
N	Pt0-2	F	41	3.9	3	ned			N	112
N	Pt0-3	M	56	2.2	3	ned			N	97
N	Pt0-4	F	73	4.9	2	ned			N	63
N	Pt0-5	F	29	5.3	3	ned			N	67
N	Pt0-6	M	54	3.2	2	ned			N	54
N	Pt0-7	F	30	6.0	3	ned			N	58
N	Pt0-8	F	46	7.0	3	ned			N	25
Y	Pt1-1	F	68	5.7	3	pd	42	Lung	N	93
Y	Pt1-2	M	21	6.5	3	pd	2	Lung, Lymph nodes, liver	Y	11
Y	Pt1-3	M	57	6.8	3	pd	18	Lung, Lymph nodes, liver	N	40

^a^ ned, no evidence of disease; pd, progression of the disease; ^b^ recurrence-free time was defined as the time from nephrectomy until the first time that the patient was known to have recurrence; ^c^ survival time was defined as the time from nephrectomy until death or the last time that the patient was known to be alive; WHO, World Health Organization; ISUP, International Society of Urologic Pathologists.

**Table 2:** Significantly upregulated/downregulated genes in clinical T1N0M0 Xp11.2 translocation renal cell carcinoma with versus without aggressive characteristics (recurrence or cancer-specific death).

Upregulated				Downregulated			
Gene Symbol	Gene Name	Log_2_ Fold Change (Aggressive/Non-Aggressive)	# Of Related Pathways	Gene Symbol	Gene Name	Log_2_ Fold Change (Aggressive/Non-Aggressive)	# Of Related Pathways
*KRT10*	Keratin 10	2.09	1	*ZNF662*	Zinc Finger Protein 662	−2.56	1
*PIK3R2*	Phosphoinositide-3-Kinase Regulatory Subunit 2	2.22	11	*RARB*	Retinoic Acid Receptor Beta	−2.76	1
*PCYT2*	Phosphate Cytidylyltransferase 2, Ethanolamine	2.37	1	*ATP1B2*	ATPase Na+/K+ Transporting Subunit Beta 2	−3.36	2
*GALE*	UDP-Galactose-4-Epimerase	2.48	2	*PDE7B*	Phosphodiesterase 7B	−3.73	1
*GPX1*	Glutathione Peroxidase 1	2.55	2	*KCNMB4*	Potassium Calcium-Activated Channel Subfamily M Regulatory Beta Subunit 4	−5.54	1
*MIF*	Macrophage Migration Inhibitory Factor	2.64	1	*NAT2*	N-Acetyltransferase 2	−6.45	3
*SHC1*	SHC Adaptor Protein 1	2.66	1	*OR56A5*	Olfactory Receptor Family 56 Subfamily A Member 5	−6.91	1
*DTYMK*	Deoxythymidylate Kinase	2.71	1	*OR2T7*	Olfactory Receptor Family 2 Subfamily T Member 7	−7.47	1
*ENO1*	Enolase 1	2.93	4	*OR10A2*	Olfactory Receptor Family 10 Subfamily A Member 2	−8.75	1
*TALDO1*	Transaldolase 1	3.00	3	*ZNF479*	Zinc Finger Protein 479	−9.17	1
*GSS*	Glutathione Synthetase	3.30	2	*GK2*	Glycerol Kinase 2	−9.30	1
*TYMP*	Thymidine Phosphorylase	3.42	2	*OR6C70*	Olfactory Receptor Family 6 Subfamily C Member 70	−9.67	1
*HLA-DRB5*	Major Histocompatibility Complex, Class II, DR Beta 5	9.81	4	*OR10H2*	Olfactory Receptor Family 10 Subfamily H Member 2	−9.89	1
*PTGDS*	Prostaglandin D2 Synthase	23.73	1	*OR2L3*	Olfactory Receptor Family 2 Subfamily L Member 3	−12.53	1
				*ATP6V1C2*	ATPase H+ Transporting V1 Subunit C2	−12.56	2
				*OR5K2*	Olfactory Receptor Family 5 Subfamily K Member 2	−16.55	1

#, number.

## Data Availability

The datasets used and/or analyzed in this study are available from the corresponding author upon reasonable request.
